# Local image variance of 7 Tesla SWI is a new technique for preoperative characterization of diffusely infiltrating gliomas: correlation with tumour grade and IDH1 mutational status

**DOI:** 10.1007/s00330-016-4451-y

**Published:** 2016-06-14

**Authors:** Günther Grabner, Barbara Kiesel, Adelheid Wöhrer, Matthias Millesi, Aygül Wurzer, Sabine Göd, Ammar Mallouhi, Engelbert Knosp, Christine Marosi, Siegfried Trattnig, Stefan Wolfsberger, Matthias Preusser, Georg Widhalm

**Affiliations:** 1 High Field Magnetic Resonance Centre, Department of Biomedical Imaging and Image-Guided Therapy, Medical University of Vienna, Waehringer Guertel 18-20, 1097 Vienna, Austria; 2 Comprehensive Cancer Center, Central Nervous System Tumours Unit (CCC-CNS), Medical University of Vienna, Waehringer Guertel 18-20, 1097 Vienna, Austria; 3 Department of Health Sciences and Social Work, Carinthia University of Applied Sciences, St. Veiterstraße 47, 9020 Klagenfurt am Wörthersee, Austria; 4 Department of Neurosurgery, Medical University of Vienna, Waehringer Guertel 18-20, 1097 Vienna, Austria; 5 Institute of Neurology, Medical University of Vienna, Waehringer Guertel 18-20, 1097 Vienna, Austria; 6 Department of Radiology, Medical University of Vienna, Waehringer Guertel 18-20, 1097 Vienna, Austria; 7 Department of Internal Medicine I, Medical University of Vienna, Waehringer Guertel 18-20, 1097 Vienna, Austria

**Keywords:** 7 Tesla MRI, Diffusely infiltrating gliomas, Susceptibility-weighted imaging, Local image variance, Glioma characterization

## Abstract

**Objectives:**

To investigate the value of local image variance (LIV) as a new technique for quantification of hypointense microvascular susceptibility-weighted imaging (SWI) structures at 7 Tesla for preoperative glioma characterization.

**Methods:**

Adult patients with neuroradiologically suspected diffusely infiltrating gliomas were prospectively recruited and 7 Tesla SWI was performed in addition to standard imaging. After tumour segmentation, quantification of intratumoural SWI hypointensities was conducted by the SWI-LIV technique. Following surgery, the histopathological tumour grade and isocitrate dehydrogenase 1 (IDH1)-R132H mutational status was determined and SWI-LIV values were compared between low-grade gliomas (LGG) and high-grade gliomas (HGG), IDH1-R132H negative and positive tumours, as well as gliomas with significant and non-significant contrast-enhancement (CE) on MRI.

**Results:**

In 30 patients, 9 LGG and 21 HGG were diagnosed. The calculation of SWI-LIV values was feasible in all tumours. Significantly higher mean SWI-LIV values were found in HGG compared to LGG (92.7 versus 30.8; *p* < 0.0001), IDH1-R132H negative compared to IDH1-R132H positive gliomas (109.9 versus 38.3; *p* < 0.0001) and tumours with significant CE compared to non-significant CE (120.1 versus 39.0; *p* < 0.0001).

**Conclusions:**

Our data indicate that 7 Tesla SWI-LIV might improve preoperative characterization of diffusely infiltrating gliomas and thus optimize patient management by quantification of hypointense microvascular structures.

***Key Points*:**

• *7 Tesla local image variance helps to quantify hypointense susceptibility-weighted imaging structures*.

• *SWI-LIV is significantly increased in high-grade and IDH1-R132H negative gliomas*.

• *SWI-LIV is a promising technique for improved preoperative glioma characterization*.

• *Preoperative management of diffusely infiltrating gliomas will be optimized*.

**Electronic supplementary material:**

The online version of this article (doi:10.1007/s00330-016-4451-y) contains supplementary material, which is available to authorized users.

## Introduction

Diffusely infiltrating gliomas are the most frequent primary brain tumours in adults [1]. According to the current World Health Organization (WHO) criteria, the histopathological spectrum of diffusely infiltrating gliomas ranges from slowly growing tumours (low-grade gliomas = LGG; WHO grade II) to highly malignant neoplasms (high-grade gliomas = HGG; WHO grades III and IV) [2]. Following neurosurgical resection or biopsy of HGG, immediate postoperative treatment with radio- and/or chemotherapy is crucial, while in most LGG maximal safe tumour resection without initial postoperative therapy is performed [3–5]. LGG typically show malignant progression to HGG within several years, where the formation of pathological microvessels by neo-angiogenesis represents one of the key steps [2, 6]. Thus, the detection of these pathological microvascular structures is crucial for histopathological differentiation of LGG from HGG: while in LGG (WHO grade II) angiogenic features are typically absent, glioblastoma multiforme (GBM; WHO grade IV), the most common and malignant form of glioma, is characterized by the presence of pathognomonic microvascular proliferates [2, 6].

Nowadays, new molecular markers have been introduced that are capable of further refining the classification of gliomas into distinct subtypes [7, 8]. Most notably, presence of the isocitrate dehydrogenase 1 (IDH1) mutation was shown to be associated with WHO grade II/III gliomas and secondary GBM as well as a significantly longer progression-free and overall survival [7–10]. By far the most common IDH1 mutation involves the amino acid 132 at exon 4 (IDH1-R132H) [11]. More and more, molecular markers such as the IDH1 mutational status are increasingly incorporated in clinical decision making in addition to the tumour grade. Furthermore, it has been recently demonstrated that IDH1 mutant gliomas particularly profit from aggressive tumour resections [12, 13]. Similarly to different microvascular patterns in gliomas of various grades of malignancy, neo-angiogenesis, and thus formation of pathological microvessels was found to be associated with IDH1/2 mutational status with increased neo-angiogenesis in IDH1/2 wild-type gliomas and inhibition of neo-angiogenesis in IDH1/2 mutant tumours [14]. Thus, reliable identification of these pathological microvascular structures is essential for preoperative glioma characterization to plan the appropriate surgical strategy and postoperative therapy, as well as assessment of the individual patient prognosis.

Routinely, preoperative neuroradiological differentiation between LGG and HGG is mainly based on the type of MRI contrast-enhancement (CE) on T1-weighted images [15]. Pathological microvascular proliferation of HGG is associated with impairment of the blood-brain barrier resulting in visible leakage of contrast medium (CM) into the brain tumour tissue on MRI [15]. Thus, the presence of significant CE is usually considered as indicator of HGG, while LGG generally lack unequivocal CE [2, 15, 16]. However, the type of CE on MRI represents only an indirect sign of microvascular alterations. Furthermore, assessment of gliomas by conventional contrast-enhanced MRI is associated with specific drawbacks: (1) Gliomas frequently show a diffuse pattern of CE that is not able to differentiate specific structures such as the tumour microvascularity [17, 18]. (2) Up to 50 % of HGG lack distinct CE on MRI mainly due to insufficient extravasation of CM that does not allow reliable visual detectability [19, 20]. (3) Additionally, in a fraction of patients the administration of CM is not feasible due to CM specific contraindications [21]. In recent years, also “advanced imaging techniques” are increasingly applied in addition to standard contrast-enhanced MRI such as diffusion weighted imaging (DWI), contrast and non-contrast perfusion MRI and MR spectroscopy to optimize preoperative glioma grading [22, 23]. For preoperative identification of the IDH1 mutational status, reliable techniques are currently not available in routine clinical practice so far although promising approaches such as MR spectroscopy of 2-hydroxyglutarate were described [24]. Consequently, new reliable methods for direct visualization of pathological microvascularity in gliomas must be developed for preoperative estimation of the tumour grade and IDH1 mutational status.

Susceptibility-weighted imaging (SWI) was reported so far for different indications such as visualization of brain vascularization, arteriovenous and cavernous malformations, multiple sclerosis lesions, cerebral abscesses, stroke, brain function, and brain tumours [17, 25–32]. Recently, it was found that SWI hypointensities within gliomas are mostly caused by tumour microvascularity, blood deposits, and calcifications that are frequently observed in oligodendrogliomas and that application of ultra high-field MRI using 7 Tesla SWI provides high spatial resolution to visualize such pathological intratumoural alterations [33–35]. To date, however, no reliable methods for precise quantification of these hypointense SWI structures have been established in clinical routine. Here, we introduce the SWI based local image variance (LIV) to quantify SWI hypointensities. The LIV is a measure of image variation in the vicinity of a pixel, which is in SWI increased in cases of a high density of blood vessels or microbleeds represented as signal loss on SWI images.

The aim of this study was thus to investigate the value of LIV as a new technique for quantification of SWI hypointensities at 7 Tesla for preoperative non-invasive assessment of the tumour dignity in diffusely infiltrating gliomas. Hence, we prospectively analyzed a series of adult glioma patients and compared 7 Tesla SWI-LIV values to the WHO tumour grade, IDH1-R132H mutational status, and type of CE on MRI.

## Materials and methods

### Patients

In this study, adult patients scheduled for neurosurgical resection or biopsy of a radiologically suspected diffusely infiltrating glioma (WHO grades II-IV) were prospectively recruited between 2009 and 2015. This was a prospective, single arm, non-randomized study. In each patient, 7 Tesla MRI was performed prior to the neurosurgical procedure in addition to standard imaging for calculation of SWI-LIV values. None of the patients received previous surgery, chemo- and/or radiotherapy for a brain tumour prior to inclusion into this study. The protocol of this study was approved by the local institutional review board and written informed consent was obtained from all patients. Patient characteristics are provided in Table 1.

**Table 1:** Patients’ characteristics

		*n*	*%*
number of patients		30	(100)
gender	male : female	1 : 1.5	
age (years)	median (range)	51 years (21 - 78)	
localization	frontal	10	(34)
	insular	5	(17)
	temporal	4	(14)
	parietal	3	(10)
	central	3	(10)
	occipital	1	(3)
	trigonal	1	(3)
	brainstem	1	(3)
	thalamus	1	(3)
	corpus callosum	1	(3)
MRI contrast - enhancement			
*significant*	ring-like	11	(36)
	nodular	2	(7)
*non-significant*	none	12	(40)
	patchy/faint	3	(10)
	focal	2	(7)
type of surgery	resection	19	(64)
	stereotactic biopsy	10	(33)
	open biopsy	1	(3)
target for iOP tumour sampling			
	MR CE	13	(43)
	PET	15	(50)
	CSI	2	(7)
diagnosis			
*HGG*	glioblastoma *(WHO grade IV)*	12	(40)
	gliosarcoma *(WHO grade IV)*	1	(3)
	anaplastic astrocytoma *(WHO grade III)*	5	(18)
	anaplastic oligodendroglioma (*WHO grade III*)	2	(7)
	mixed oligoastocytoma (*WHO grade III*)	1	(3)
*LGG*	oligodendroglioma *(WHO grade II)*	4	(13)
	diffuse astrocytoma *(WHO grade II)*	4	(13)
	mixed oligoastocytoma (*WHO grade II*)	1	(3)
IDH1 mutational status			
	IDH1-R132H negative tumour	15	(50)
	IDH1-R132H positive tumour	15	(50)

*CE* contrast - enhancement; *CSI* chemical shift imaging; *HGG* high-grade gliomas; *iOP* intraoperative, *LGG* low-grade gliomas, *MR* magnetic resonance imaging, *PET* positron emission tomography, *WHO* World Health Organization; *IDH1* isocitrate dehydrogenase 1

### Preoperative imaging

Routinely, preoperative MRI was performed on a 3 Tesla scanner (Tim Trio, Siemens, Erlangen, Germany) in all patients as described previously [36, 37]. According to the type of CE on MRI, we distinguished gliomas with significant (ring-like or nodular) or non-significant CE (none, patchy/faint or focal) [36–39]. In all patients with non-significant CE, we performed an additional positron emission tomography (PET) using amino acid tracer (^11^C-methionine or ^18^F-fluoro-ethyl-L-tyrosine) and/or MRI spectroscopy, chemical shift imaging (CSI), for detection of a potential metabolic “hotspot” for tissue sampling [38, 39].

### 7 Tesla imaging

For the current study, we additionally performed a preoperative 7 Tesla MRI (Magnetom©, Siemens Healthcare, Erlangen, Germany) in all patients. In this study, only patients were included in which a 24- or a 32-channel radio frequency coil (Nova Medical, Wilmington, DE, USA) was applied in the frame of the 7 Tesla MR investigations. All patients underwent the same protocol for image acquisition including a T1-weighted and a SWI sequence: (1) T1-weighted data were acquired with and without CM administration (Multihance; 0.2 mL per kilogram of body weight) using a magnetization prepared rapid gradient echo (MPRAGE) sequence with the following parameters: image-matrix = 320 × 320 pixels; resolution = 0.75 × 0.72 × 0.7 mm; slices = 208; parallel imaging factor = 2, TR/TI/TE 3800/1700/3.55 ms, acquisition time = 10:29 min. (2) Between the T1-weighted measurements a SWI sequence with a TE of 15 ms was performed to acquire SWI data (before CM administration). Further sequence parameters were: TR = 28 ms; image-matrix = 704 × 704 pixels; slices = 96; parallel imaging factor = 2, acquisition time = 10:18 min, resolution = 0.3 × 0.3 × 1.2 mm. It is of note, that SWI was acquired before CM administration. Phase filtering and SWI image processing was performed directly by the Siemens SWI sequence.

### Definition of regions of interest

To define regions of interest (ROI), manual tumour segmentation was performed using contrast-enhanced T1-weighted sequences or T2-weighted/FLAIR images (3 Tesla MRI data) and the StealthViz neurosurgical planning software (Version 1.2, Medtronic, Louisville, CO, USA). Referring to the Response Assessment in Neuro-Oncology (RANO) criteria, the extension of the glioma was defined by the contrast-enhancing area on T1-weighted MR images in suspected HGG with significant CE and by the non-enhancing hyperintense lesion on T2-weighted/FLAIR sequences in suspected LGG with no significant CE [40]. As LIV values describe the image heterogeneity in neighbouring voxels, care was taken not to include LIV values derived from heterogeneities caused by other sources than pathological intratumoral alterations such as the brain surface, neighbouring blood vessels or the ventricles. In such cases the initially segmented tumour ROI was adjusted to exclude such confounding values.

### SWI-LIV calculation

First of all, the segmented ROIs, the 3 Tesla T1-weighted as well as the 7 Tesla T1-weighted and SWI data were converted to the medical imaging network common data (MINC) format (see also Supplementary Figure 1) [41]. The MINC format and its corresponding toolbox were also used for image processing. Before the LIV calculation, image pre-processing was performed in which the SWI data were intensity-corrected using histogram spline sharpening and afterwards image intensity was rescaled in the arbitrary range between 0 and 100 [42]. These steps are crucial in order to avoid differences in variance based on different image intensities. In the next step, the 7 Tesla SWI-LIV was calculated by using a modified version of the Steiner translation theorem [43]:

$$ LIV=G\left({X}^2\right)-{\left[G(X)\right]}^2 $$

where *X* is the pre-processed SWI image and *G* represents Gaussian low pass filtering, where a kernel with a full width at half maximum (FWHM) of 3 mm was found the most appropriate for the current data.

**Fig. 1:**
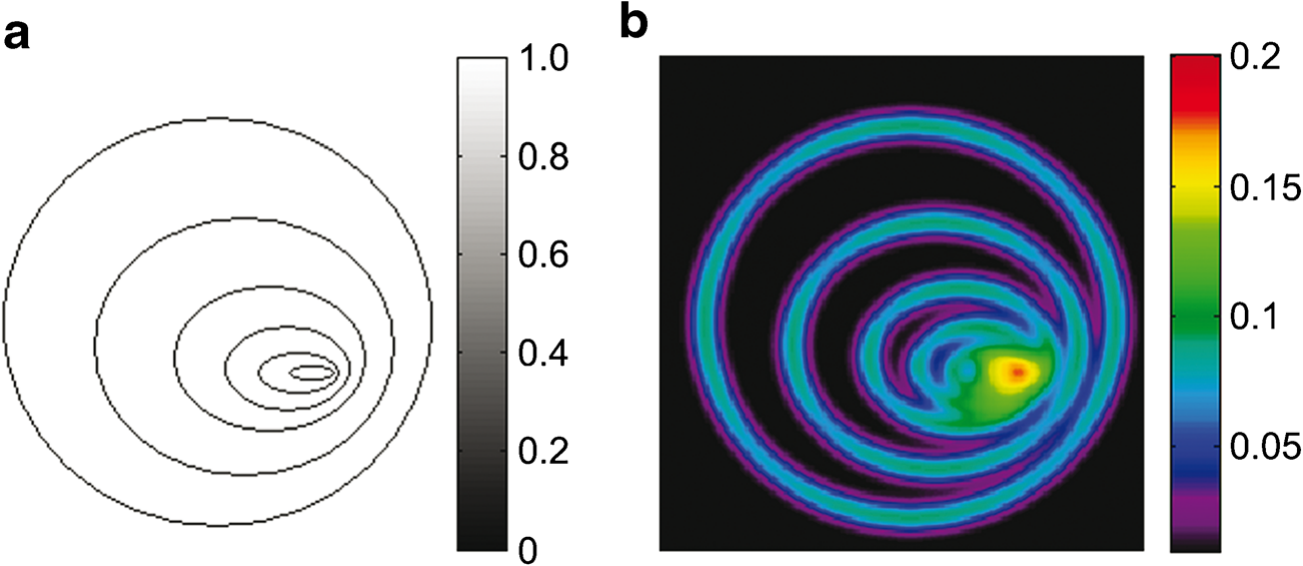
Illustration of the SWI - local image variance (LIV) calculation. (A) The left image shows a simulation of blood vessels (hypointense structures on a hyperintense background) as typically detected by SWI. As illustrated in this image, the density of these simulated blood vessels increases from the left to the right. Such an increasing density of blood vessels is typically observed in high-grade gliomas. (B) The right image illustrates the corresponding colour-coded SWI-LIV map, where LIV values increase with the density of blood vessels. The LIV values reach their maximum in the area with the highest density of the blood vessels. Note, the simulated SWI image (left image) is a binary image and includes only the values 0 and 1, resulting in SWI-LIV values that are not comparable to patient data

Figure 1 illustrates the SWI-LIV on a simulated SWI image including hypointense structures with varying density. To quantify hypointensities (e.g. microvascularity) within patient data, the segmented tumour ROIs were linearly transformed from the 3 Tesla into the 7 Tesla image space using T1-weighted images for registration. The mean SWI-LIV values for the individual tumours were afterwards calculated using the transformed tumour ROIs (see Figs. 3d, 4d, and 5d).

### Neurosurgical procedures

During all glioma resections or biopsies a navigation system (Stealth Station Cranial Mach 5 or S7; Medtronic, CO, USA) with integrated T1-weighted contrast-enhanced MR images was used. In gliomas with non-significant CE, additional T2-weighted/FLAIR images and PET imaging or CSI were co-registered with T1-weighted MRI. To avoid acquisition of non-representative tumour tissue and thus histopathological undergrading, we performed separate navigation-guided tissue sampling from the area of unequivocal CE (gliomas with significant CE) or the PET/CSI hotspot (gliomas with non-significant CE) in all tumour resections or biopsies as previously described [37, 38, 44]. For intraoperative confirmation of the correct tissue target area (CE, PET, or CSI) independent of brain-shift, we additionally used 5-aminolevulinic acid (5-ALA) during resections or biopsies as described previously in the vast majority of cases (*n* = 29 of 30 patients) [36, 44].

### Histopathology

Histopathological tumour diagnoses were made according to the current edition of the WHO criteria [2]. The IDH1 mutational status was analyzed by immunohistochemistry using the IDH1-R132H mutation-specific antibody (anti-human IDH1-R132H; pH6, 1:30, Dianova, Germany). The neuropathologists were blinded to the MR-based SWI pattern of all gliomas.

### Glioma grading with standard MRI sequences and SWI-LIV

To investigate the value of SWI-LIV for glioma grading, we compared this technique for preoperative estimation of the correct WHO grade (LGG versus HGG) with standard MR sequences. For this purpose, an experienced neuroradiologist graded all tumours in LGG and HGG using standard 3 Tesla MR sequences (T1-weighted sequences with and without CM and T2-weighted images). For comparison, all tumours were additionally graded using the SWI-LIV values, where we applied a SWI-LIV cutoff value of 35 for differentiation of LGG and HGG. This cut off value was defined prior to this analysis by comparing SWI-LIV values between data sets of LGG and HGG. Consequently, all tumours with SWI-LIV ≤ 35 were classified as LGG and all gliomas with SWI-LIV > 35 as HGG in this comparative analysis.

### Statistical analysis

For statistical analyses, SPSS® version 23.0 software (SPSS Inc., Chicago, IL, USA) was applied. We used a non-parametric Mann-Whitney U test for comparison of SWI-LIV with the WHO tumour grade, IDH1-R132H mutational status and type of MRI CE. Furthermore, we applied the Spearman’s rank correlation coefficient to analyze the correlation between SWI-LIV and the WHO tumour grade. A *p*-value of <0.05 was considered significant.

## Results

Altogether 30 patients (18 women and 12 men) with a median age of 51 years (range 21 to 78 years) were included. In 19 patients a tumour resection (64 %), 10 cases a stereotactic biopsy (33 %), and one patient an open biopsy (3 %) was performed. Histopathological WHO diagnosis revealed a LGG in nine cases and a HGG in 21 patients. Further patient characteristics are summarized in Table 1.

### SWI-LIV and WHO tumour grade

**Fig. 2:**
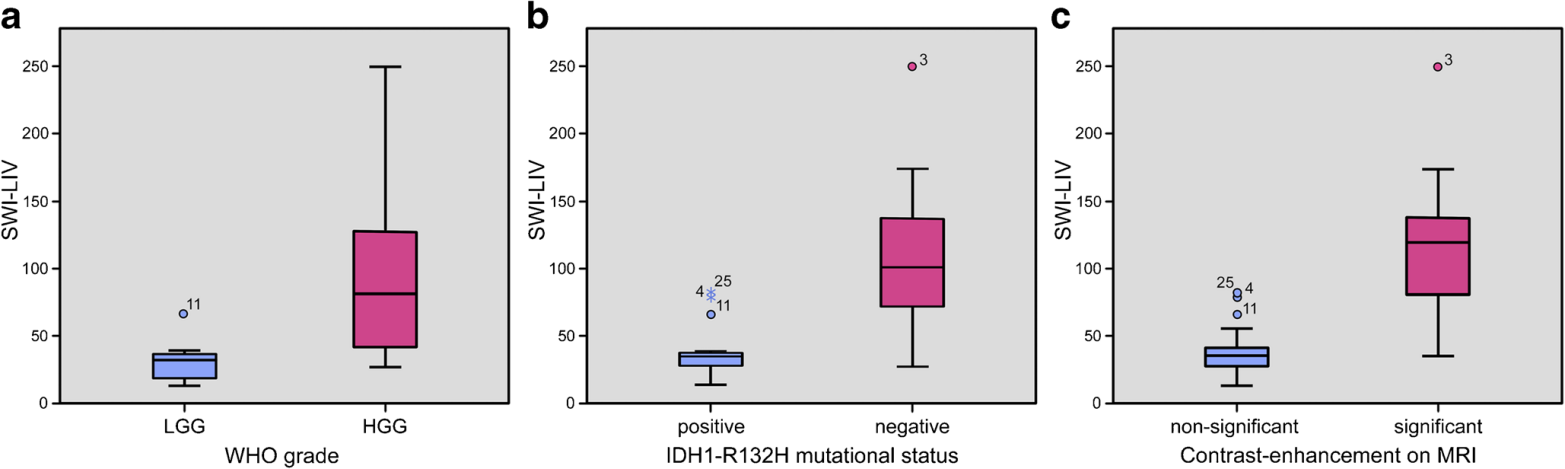
Boxplot diagrams for comparison of SWI-LIV values with tumour grade, IDH1-R132H mutational status and type of CE on MRI. (A) SWI-LIV and tumour grade: The mean SWI-LIV was significantly higher in HGG as compared to LGG (92.7 versus 30.8; *p* < 0.0001). (B) SWI-LIV and IDH1-R132H mutational status: The mean SWI-LIV was significantly higher in IDH1-R132H negative gliomas as compared to IDH1-R132H positive gliomas (109.9 versus 38.3; *p* < 0.0001). (C) SWI-LIV and type of CE on MRI: The mean SWI-LIV was significantly higher in gliomas with significant CE as compared to non-significant CE (120.1 versus 39.0; *p* < 0.0001)

**Fig. 3:**
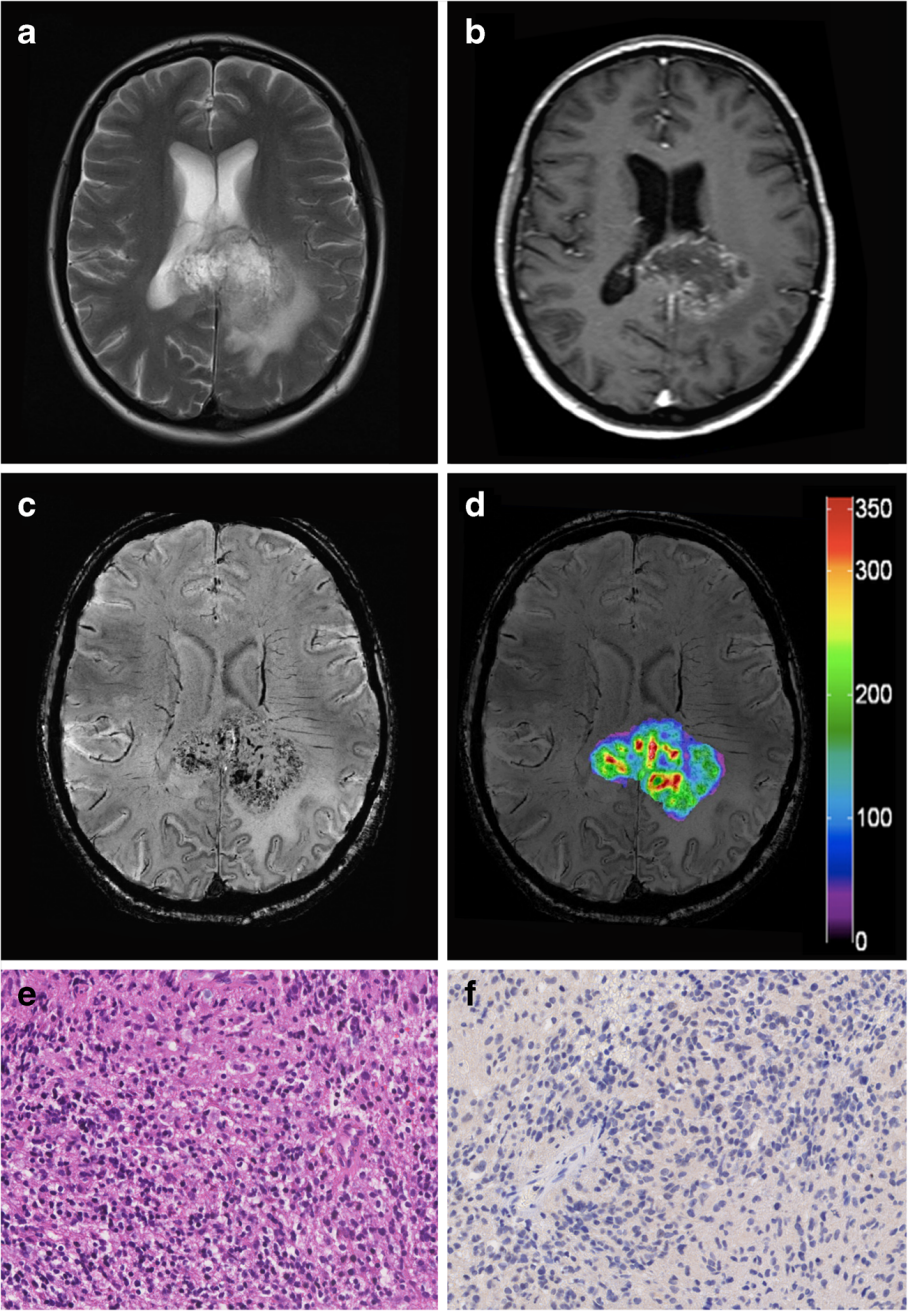
Representative case of a HGG with negative IDH1-R132H mutational status. (A) Preoperative axial T2-weighted MR images and (B) contrast-enhanced T1-weighted sequences show a hyperintense lesion in the posterior portion of the corpus callosum with significant (ring-like) CE. (C) 7 Tesla SWI detects markedly increased intratumoral hypointense structures. (D) The 7 Tesla SWI is overlaid with the corresponding SWI-LIV map. The colour-coded SWI-LIV map in this glioma shows high values (mean SWI-LIV: 135.7) for areas with a high variability that correspond probably to intratumoral regions with high amounts of pathological microvessels and microhaemorrhages. It is of note that the area, where LIV values are shown, represents the segmented tumour ROI. (E) Histopathological examination reveals a HGG (GBM; WHO grade IV) with (F) negative IDH1-R132H mutational status. Original magnification of the H&E stain (E) and IDH1-R132H stain (F) is × 200

**Fig. 4:**
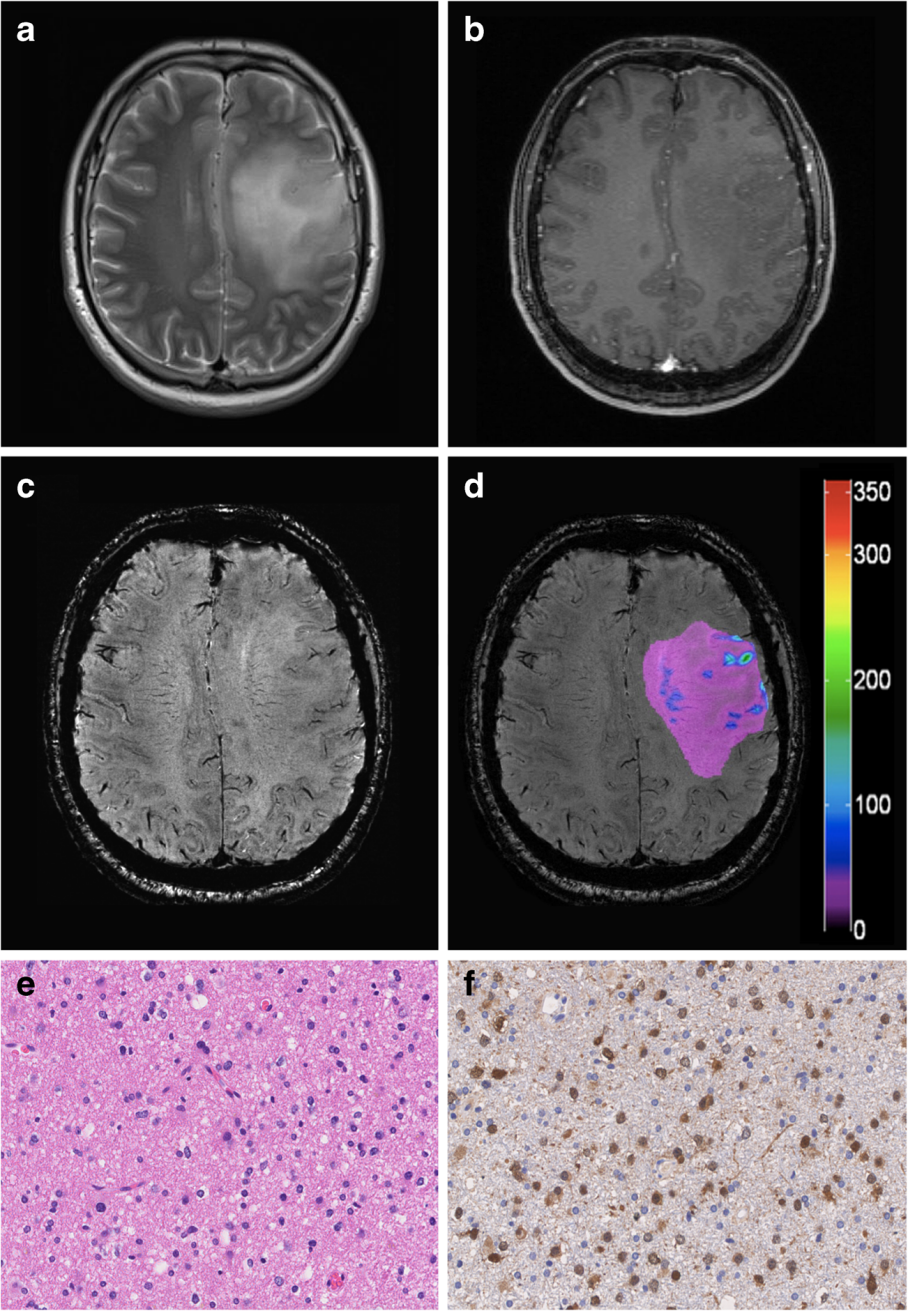
Representative case of a LGG with positive IDH1-R132H mutational status. (A) Preoperative axial T2-weighted MR images and (B) contrast-enhanced T1-weighted sequences demonstrate a left frontal hyperintense lesion with non-significant (patchy/faint) CE. (C) 7 Tesla SWI detects only very few hypointense structures and (D) the corresponding colour-coded SWI-LIV map shows low values in the tumour ROI (mean SWI-LIV: 32.2). (E) Histopathological examination reveals a LGG (diffuse astrocytoma WHO grade II) with (F) positive IDH1-R132H mutational status. Original magnification of the H&E stain (E) and IDH1-R132H stain (F) is × 200

Calculation of SWI-LIV maps was feasible in all patients. The mean SWI-LIV value of all gliomas was 74.1 with a standard deviation (SD) of 56.2. We found a significantly higher mean SWI-LIV in HGG (92.7; SD = 57.2) compared to LGG (30.8; SD = 16.3; *p* < 0.0001) and a significant correlation of SWI-LIV values with the WHO tumour grade (R = 0.81; *p* < 0.0001). See also Table 2, Figs. 2, 3, and 4.

**Table 2:** Correlation of SWI-LIV with the WHO tumour grade, IDH1 mutational status, and type of contrast-enhancement on MRI

	*n*	SWI-LIV		*p-value*
		*mean*	*SD*	
WHO grade				
*LGG*	*9*	30.8	16.3	*<0.0001*
*HGG*	*21*	92.7	57.2	
IDH1-R132H mutational status				
*positive*	*15*	38.3	21.1	*<0.0001*
*negative*	*15*	109.9	57.9	
CE on MRI				
*non-significant*	*17*	39.0	20.4	*<0.0001*
*significant*	*13*	120.1	55.2	

*CE* contrast enhancement, *HGG* high-grade gliomas, *IDH1* isocitrate dehydrogenase 1, *LGG* low-grade gliomas, *MRI* magnetic resonance imaging, *SD* standard deviation, *SWI* susceptibility weighted imaging, *WHO* World Health Organization

### SWI-LIV and IDH1-R132H mutational status

IDH1-R132H was not detected by immunohistochemistry in 15 gliomas (12 WHO grade IV and three WHO grade III gliomas), while a positive IDH1-R132H status was observed in the remaining 15 gliomas (nine WHO grade II, five WHO grade III, and one WHO grade IV gliomas). The mean SWI-LIV in IDH1-R132H negative gliomas (109.9; SD = 57.9) was significantly higher compared to IDH1-R132H positive gliomas (38.3; SD = 21.1; *p* < 0.0001). See also Table 2, Figs. 2, 3, and 4.

### SWI-LIV and type of contrast-enhancement

Significant CE on preoperative MRI was detected in 13 gliomas (11 ring-like and two nodular CE) and non-significant CE was observed in 17 cases (12 none, three patchy/faint, and two focal CE). We found significantly higher mean SWI-LIV values in gliomas with significant CE (120.1; SD = 55.2) compared to non-significant CE (39.0; SD = 20.4; *p* < 0.0001). See also Table 2, Figs. 2, 3, and 4.

### Comparison of glioma grading with standard MRI sequences and SWI-LIV

**Fig. 5:**
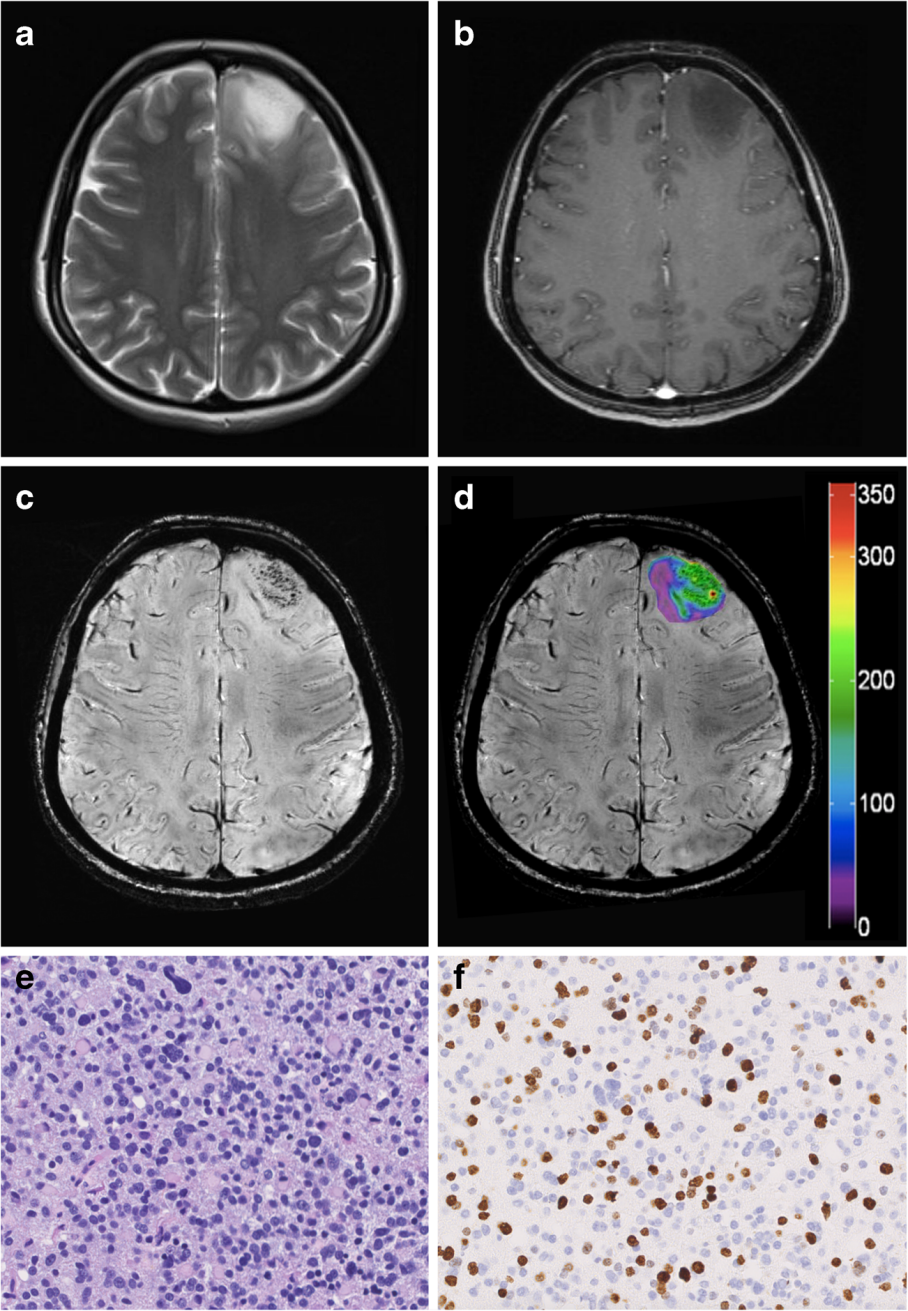
Preoperative identification of early malignant transformation in an initially suspected LGG on conventional MRI. (A) Preoperative axial T2-weighted MR images and (B) contrast-enhanced T1-weighted sequences reveal a left frontal hyperintense lesion with non-significant (none) CE. (C) Although no CE is visible on conventional MRI, 7 Tesla SWI depicts markedly increased intratumoural hypointense structures and (D) the corresponding colour-coded SWI-LIV map shows high values in the tumour ROI (mean SWI-LIV: 82.0). (E) Histopathological examination depicts already a HGG (anaplastic oligodendroglioma WHO grade III) with a (F) markedly increased proliferation rate (MIB-1 labelling index: 30 %). Original magnification of the H&E stain (E) and anti–Ki 67 stain (F) is × 200

According to standard 3 Tesla MR sequences, an experienced neuroradiologist correctly graded six out of nine LGG and 14 out of 21 HGG. In contrast, SWI-LIV correctly graded six out of nine LGG and 19 out of 21 HGG by using a SWI-LIV cutoff value of 35. Therefore, the SWI-LIV value correctly graded five more tumours (five HGG) than the neuroradiologist (see also Fig. 5).

## Discussion

Herein, we describe a new technique for quantitative analysis of hypointense SWI structures in diffusely infiltrating gliomas. In this prospective study using 7 Tesla MRI, we found significantly higher SWI-LIV values in HGG compared to LGG, IDH1-R132H negative compared to IDH1-R132H positive gliomas and tumours with significant CE compared to non-significant CE on MRI.

### SWI for visualization of tumour microvascularity and current drawbacks

SWI is generally capable to visualize normal vascular brain structures as well as pathological brain microvascularity and is thus increasingly applied additionally to conventional MRI [18, 33, 34]. Therefore, the use of SWI seems to be of special value for characterization of tumour microvascularity to improve preoperative glioma grading. In this sense, Li et al. detected a significantly higher number of small vessels and micro-haemorrhage volume in high-grade compared to low-grade astrocytomas using 3 Tesla SWI [17]. Hori et al. observed significantly higher SWI hypointensity ratios at 3 Tesla MRI in HGG compared to LGG by semiquantitative assessment [32]. Moreover, further studies using semiquantitative analyses of 1.5 or 3 Tesla SWI data found a higher degree of the “intratumoral susceptibility signal intensity” in HGG compared to LGG [31, 45, 46]. Additionally, Moenninghoff et al. observed an increasing tumour microvascularity in single cases of high-grade compared to low-grade astrocytomas using 7 Tesla SWI [47]. These authors also found that 7 Tesla MRI is capable to display the tumour microvascularity more detailed compared to images derived from lower field strengths [47]. In these studies, different methods have been applied for analysis of intratumoral SWI hypointensities that are, however, mainly based on visual and semiquantitative assessment and are thus subjective and observer dependent. So far, no reliable methods for precise quantification of hypointense SWI structures have been established in clinical routine. Consequently, the development of clinically reliable techniques for precise quantification of intratumoral SWI hypointensities that are more objective is of major importance.

### Present study: SWI-LIV and WHO tumour grade

We introduce herein a new technique for quantification of SWI hypointensities by using the so-called local image variance. In this study, SWI was performed on 7 Tesla with the advantage of a higher contrast and resolution for improved visualization of tumour microvascularity compared to lower field strengths. By clinical application of this technique, we found a significantly higher mean SWI-LIV in HGG compared to LGG and observed a strong correlation of SWI-LIV with the tumour grade. Compared to semiquantitative parameters, the SWI-LIV technique allows a precise quantitative analysis of SWI hypointensities that correspond mainly to tumour microvascularity and microhemorrhages. Thus, the SWI-LIV technique represents a new clinically reliable and objective technique for improved preoperative glioma grading. Consequently, clinical application of SWI-LIV will in future improve planning of the appropriate postoperative therapy and assessment of individual patient prognosis.

### SWI-LIV and IDH1 mutational status

The preoperative knowledge of the IDH1 status is of major interest for further patient management/surgical strategy since it was shown in a recent study that the IDH1 mutational status has a crucial impact on the surgical benefit: While patients with IDH1 wild-type malignant gliomas do not profit from further removal of the non-enhancing tumour in addition to the enhancing tumour, this surgical strategy results in a significantly prolonged overall survival in patients with IDH1 mutant tumours [12, 13]. Interestingly, we observed a significantly higher mean SWI-LIV in IDH1-R132H negative compared to IDH1-R132H positive gliomas. Therefore, the SWI-LIV technique might in future also preoperatively identify candidates that particularly profit from aggressive glioma resections. To our knowledge, this is the first systematic analysis of SWI in the subgroup of IDH1-R132H negative and positive diffusely infiltrating gliomas. This is in accordance with a recent study by Kickingereder et al. that reported a markedly higher relative cerebral blood volume (rCBV) in IDH1/2 wild-type gliomas compared to IDH1/2 mutant gliomas [14]. Similarly, Lee et al. found that IDH1 negative gliomas were characterized by higher 90th percentile normalized cerebral blood volume (nCBV) values as well as a steeper slope in histograms including cumulative nCBV data [48]. Accordingly, Yamashita et al. observed a significantly increased absolute tumour blood flow measured by arterial spin-labelling/perfusion-MRI and tumour necrotic area on MRI in IDH1 wild-type compared to IDH1 mutant GBM [49]. These data including our own findings demonstrate that assessment of SWI and perfusion values is a promising method not only for preoperative determination of the correct WHO tumour grade, but also the IDH1 mutational status in gliomas. However, our initial observation should be confirmed also in future studies and SWI values should be correlated with perfusion parameters. One current shortcoming is that SWI-LIV values can be increased in case of a HGG or an IDH1-R132H negative glioma and thus the SWI-LIV technique alone cannot differentiate between these two aspects. To overcome this limitation, the combination of SWI-LIV with other imaging sequences such as MR spectroscopy might be considered in future to distinguish between IDH1-R132H positive and negative gliomas independent of the WHO tumour grade.

### SWI-LIV and CE on MRI

Finally, we observed significantly higher mean SWI-LIV values in gliomas with significant CE compared to non-significant CE on preoperative MRI. Consequently, the application of SWI might be of special value in patients with contraindications to CM administration such as severe renal insufficiency [21]. Furthermore, SWI represents a powerful method for visualization of the internal architecture of brain tumours such as the tumour microvascularity that provides complementary information to conventional MRI [17, 18, 50]. In this sense, SWI-LIV maps might identify also areas of increased tumour microvascularity corresponding to early malignant transformation of initially LGG prior to the presence of significant CE on MRI in selected cases (see Fig. 5). Interestingly, five HGG of this study were only detected by the SWI-LIV technique, but not by an experienced neuroradiologist analyzing standard MR sequences including contrast-enhanced images.

### Specific characteristics of the SWI-LIV technique

In order to calculate robust and comparable SWI-LIV maps, it is crucial to acquire all SWI images with the same imaging parameters. Additionally, image pre-processing is important to remove low-frequency intensity gradients, which are common in ultra high-field images. Also, initial image intensities can differ between MRI measurements. Consequently, correction of intensity difference is essential to avoid a LIV offset. As we used two different coils (24- and 32-channel coils) for 7 Tesla MRI, we evaluated SWI-LIV values in normal appearing white matter (NAWM) regions at the level of the lateral ventricles in all data sets and found no significant difference in these ROIs between the two different coils. Therefore, both coils can be applied for the calculation of SWI-LIV values at 7 Tesla MRI according to our experience. Although our SWI-LIV maps were based on 7 Tesla data, the SWI-LIV technique is by no means limited to 7 Tesla. To demonstrate the feasibility of the SWI-LIV technique also in MR investigations with lower field strengths, we performed a SWI-LIV analysis in two representative patients (one LGG and one HGG) of our study in which 3 Tesla SWI data sets were available. According to this analysis, we found that the calculation of SWI-LIV is in principle also feasible in MR investigations with lower field strengths. However, the value of the SWI-LIV technique for analysis of data derived from scanners with lower field strengths that are more widely available should be investigated in future. Another future step should be the introduction of a cutoff value in order to automatically classify tumours as either LGG or HGG. With a SWI-LIV cutoff value of 35, we were able to estimate the correct glioma grade (LGG versus HGG) more frequently with the SWI-LIV technique compared to the judgment of an experienced neuroradiologist using standard MR sequences. However, the SWI-LIV cutoff value is largely dependent on the imaging parameters as well as the whole image processing pipeline and thus needs to be validated on a larger number of data sets and hardware configurations. Finally, since SWI data were acquired before administration of the CM, we cannot provide data in this study concerning the potential effect/influence of CM on SWI data.

## Conclusions

Our data indicate that 7 Tesla SWI-LIV is a promising method for quantification of SWI hypointensities in diffusely infiltrating gliomas. Significant differences in SWI-LIV values were found dependent on the WHO tumour grade, IDH1-R132H mutational status and type of MRI contrast-enhancement. Consequently, the clinical use of SWI-LIV might in future improve the preoperative glioma characterization and will thus optimize the patient management.

## Electronic supplementary material

Below is the link to the electronic supplementary material.

Supplementary Figure 1 SWI-LIV calculation step-by-step: (A) *Original SWI image*: As can be seen (*red ellipses*), the original SWI image is overlaid with coil related, low-frequency components. In order to get comparable SWI-LIV maps it is important to reduce these low-frequency components and to scale the image in a common intensity range, in our case 0-100. Here, low-frequency components were reduced using nu_correct and rescaling was performed using mincnorm – both techniques are part of the MINC-toolbox. (B) *Pre-processed image*: Note that the low-frequency components are reduced and that the image is scaled between 0-100. B is used to calculate LIV values using the formula as described in the manuscript. (C) *Resulting SWI-LIV map*: Note that the SWI-LIV map contains also high values for areas like the brain surface (*white ellipse*), which is not related to pathological changes. It is therefore important, that ROIs are drawn to exclude LIV values of such regions. (GIF 1077 kb)

High resolution image (TIFF 2663 kb)

## Abbreviations

5-ALA: 5-aminolevulinic acid
CE: Contrast-enhancement
CM: Contrast medium
CSI: Chemical shift imaging
DWI: Diffusion weighted imaging
FWHM: Full width at half maximum
GBM: Glioblastoma multiforme
HGG: High-grade gliomas
IDH1: Isocitrate dehydrogenase 1
LGG: Low-grade gliomas
LIV: Local image variance
MINC: Medical imaging network common data
MPRAGE: Magnetization prepared rapid gradient echo
NAWM: Normal appearing white matter
nCBV: Normalized cerebral blood volume
PET: Positron emission tomography
RANO: Response Assessment in Neuro-Oncology
rCBV: Relative cerebral blood volume
ROI: Region of interest
SD: Standard deviation
SWI: Susceptibility-weighted imaging
WHO: World Health Organization
