# Supplementary figures and images for: Local image variance of 7 Tesla SWI is a new technique for preoperative characterization of diffusely infiltrating gliomas: correlation with tumour grade and IDH1 mutational status
Source: Eur Radiol. 2016 Jun 14;27(4):1556–67. doi: 10.1007/s00330-016-4451-y (PMC5334387; doi:10.1007/s00330-016-4451-y)

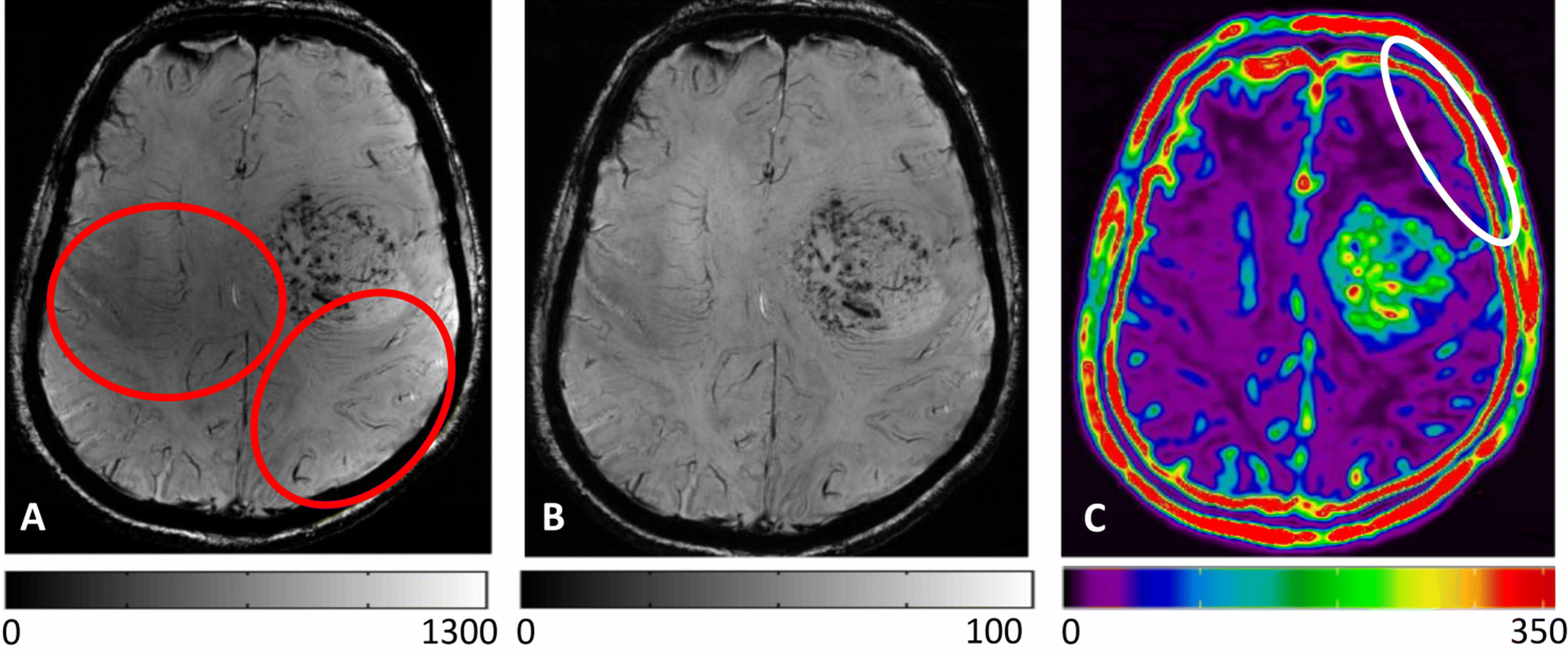

Supplement: Supplementary file 1 — SWI-LIV calculation step-by-step: (A) Original SWI image: As can be seen (red ellipses), the original SWI image is overlaid with coil related, low-frequency components. In order to get comparable SWI-LIV maps it is important to reduce these low-frequency components and to scale the image in a common intensity range, in our case 0-100. Here, low-frequency components were reduced using nu_correct and rescaling was performed using mincnorm – both techniques are part of the MINC-toolbox. (B) Pre-processed image: Note that the low-frequency components are reduced and that the image is scaled between 0-100. B is used to calculate LIV values using the formula as described in the manuscript. (C) Resulting SWI-LIV map: Note that the SWI-LIV map contains also high values for areas like the brain surface (white ellipse), which is not related to pathological changes. It is therefore important, that ROIs are drawn to exclude LIV values of such regions. (GIF 1077 kb) [file 330_2016_4451_Fig6_ESM.gif]

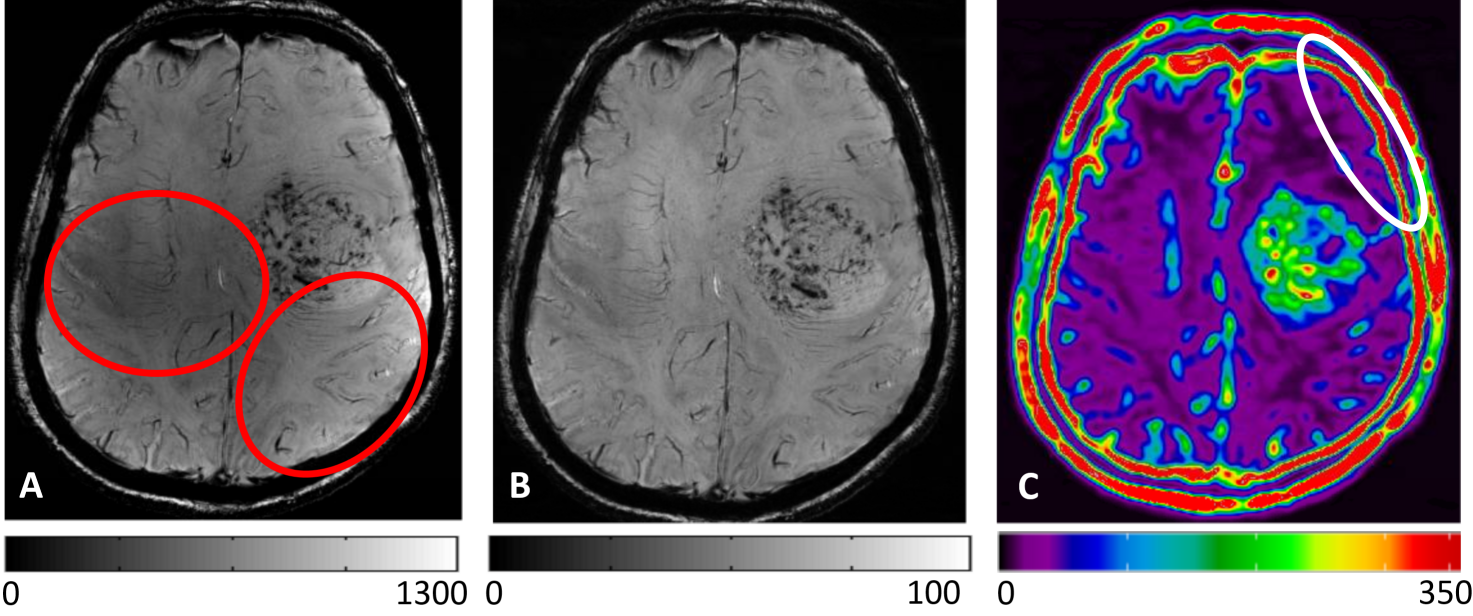

Supplement: Supplementary file 2 — High resolution image (TIFF 2663 kb) [file 330_2016_4451_MOESM1_ESM.tiff]
